# The inclusion of insect meal from *Hermetia illucens* larvae in the diet of laying hens (Hy-line Brown) affects the caecal diversity of methanogenic archaea

**DOI:** 10.1016/j.psj.2025.105037

**Published:** 2025-03-15

**Authors:** Tiziana Maria Mahayri, Jakub Mrázek, Fulvia Bovera, Giovanni Piccolo, Giovanni Andrea Murgia, Giuseppe Moniello, Kateřina Olša Fliegerová

**Affiliations:** a Laboratory of Anaerobic Microbiology, Institute of Animal Physiology and Genetics, Czech Academy of Science, 14220 Prague, Czech Republic; b Department of Veterinary Medicine, University of Sassari, Via Vienna, 2, 07100 Sassari, Italy; c Department of Veterinary Medicine and Animal Production, University of Napoli Federico II, Via F. Delpino, 1, 80137 Napoli, Italy; d External Collaborator Veterinary Practitioner, 07100 Sassari, Italy

**Keywords:** Insects, Egg layers, Methane

## Abstract

The effect of the dietary inclusion of *Hermetia illucens* larvae meal on the diversity of the methanogenic archaea in the caecum of laying hens (Hy-line Brown) was investigated using molecular methods. A total of 27 hens, selected equally for slaughter from 162 birds which were divided equally into 3 treatment groups including control group C with a diet containing corn-soybean meal and 2 experimental groups, HI25 and HI50, in which 25% and 50% of the soybean meal protein was replaced by the protein from a *Hermetia illucens* larvae meal, respectively. At 40 weeks of age, the methanogenic community of caecal content of 9 hens per group was analyzed using a 16S rRNA gene clone library. A total of 108 positive clones, 35 from the control group, 44 from the HI25 group and 29 from the HI50 group, were analyzed by Sanger sequencing. Methanomicrobiales, Methanobacteriales and Methanomassiliicoccales were the main orders found in groups C and HI25. Methanomassiliicoccales was absent in the HI50 group, which was dominated by the order Methanobacteriales. At the species level, *Methanobrevibacter woesei* was the most prevalent species in all three groups regardless of diet. Some species were found exclusively either in the control group (*Methanogenic archaeon CH1270*) or in the HI25 group (*Methanorbis furvi strain Ag1*). Methanogenic diversity was significantly lower in the HI50 group compared to the control and HI25 groups and *Methanomassiliicoccaceae archaeon DOK* was completely suppressed in HI50 group. Our preliminary results indicate that ingestion of *Hermetia illucens* larvae meal has considerable effect on the methanogenic community, promoting the abundance of *Methanobrevibacter woesei* and suppressing *Methanomassiliicoccaceae archaeon DOK* in the caeca of laying hens.

## Introduction

The poultry sector plays a crucial role in the global feed market (Mottet and Tempio, 2017). The demand for poultry meat and eggs is predicted to rise substantially in the coming years due to population growth (Farrell, 2013; Astill et al., 2020; Franzo et al., 2023). In this area, laying hens hold a pivotal position, providing a vital source of protein and essential nutrients primarily through egg production (Magdelaine, 2011; Wang et al., 2017). Eggs are not only one of the most cost-effective sources of protein but are also among the most nutritious options available (Hafez and Attia, 2020).

The gastrointestinal tract of poultry hosts a complex community of microbes (Pan and Yu, 2014; Gilroy et al., 2021), that plays crucial roles in diet digestion, nutrient absorption, immune system development and disease resistance. Alterations in this community can negatively impact feed efficiency, productivity and overall poultry health (Kohl, 2012; Shang et al., 2018). Within the gastrointestinal tract, the caecum harbors the highest microbial cell densities (Sergeant et al., 2014; Oakley et al., 2014) and serves as the primary site for microbial fermentation, contributing to the production of energetically important volatile fatty acids (Misiukiewicz et al., 2021). Caeca are mainly colonized by bacteria of the two major phyla Firmicutes and Bacteroidetes, followed by two minor phyla Actinobacteria and Proteobacteria (Rychlik, 2020). Several end products of bacterial anaerobic fermentation, especially gases H_2_, CO_2_ and acetic or formic acids are used by methanogenic archaea to generate methane, which is emitted as a by-product of enteric diet digestion to maintain hydrogen at low partial pressures to keep the thermodynamic requirements for the anaerobic fermentation processes (Mackie et al., 2024).

While the bacterial composition and diversity in different poultry species and breeds is well studied (Rychlik, 2020; Cardenas et al., 2021; Kim et al., 2023; Wang et al., 2024), information on the methanogenic microbial community are still very limited. Only a few publications have investigated methanogens in the caecal and fecal samples of chickens. Using culture-independent method, Saengkerdsub et al. (2007) described in chicken caeca 11 different sequences, however the vast majority of them (99%) was closely related to *Methanobrevibacter woesei*, a methanogen isolated also from feces of goose (Miller et al., 1986). However, studies indicate that diversity of methanogens in poultry feces is quite different. *Methanogenium* was described as a major archeon of chicken and turkey feces (Miller et al., 1986) and the very recent publication of Wang et al (2024), based on metagenome-assembled genomes, reveals new, previously unknown and much higher archaeal diversity in chicken fecal samples. In poultry research there is also virtually no information on the effect of insect meal on intestinal methanogens.

The application of insect meal in poultry diets has a growing tendency representing an innovative and promising solution to meet the growing demand for sustainable protein sources. Insect meal is known to have a positive effect on growth and health performance, meat quality and immunomodulatory properties, resulting in better disease resistence (Benzertiha et al., 2020; Elahi et al., 2022; Belhadj Slimen et al., 2023; Malematja et al., 2023). While the effect of different types of insect meals on caecal bacterial composition of poultry has been extensively studied (Józefiak et al., 2018, 2020a; Benzertiha et al., 2019a; Biasato et al., 2020; Atallah et al., 2023; Mahayri et al., 2024), its impact on methanogenic archaea remains largely unexplored. Although there is no direct evidence of the effects of insect meal on poultry methanogens, its influence on gut bacteria suggests that microbial interactions within the caecum may be affected. For instance, insect meal has been shown to increase bacterial diversity and richness, suggesting a positive effect on animal gut health (Benzertiha et al., 2019b; Józefiak et al., 2020b; Colombino et al., 2021; Vasilopoulos et al., 2024), which could indirectly impact the abundance and activity of methanogens. This lack of research on methanogens represents a critical gap, as these microorganisms play a key role in methane production, a potent greenhouse gas. Interestingly, applications of insect meal in ruminant and pig nutrition research indicates the potential to reduce methane production (Ahmed et al., 2021; Phesatcha et al., 2022). A mitigating of methane emissions may be related to changes and/or reduction in a gut methanogen population or its activity. However, similar research in poultry is lacking, despite the significant contribution of poultry production to global greenhouse gas emissions. Therefore, studying the effect of insect meal on archaea is of great importance in terms of overall efforts to reduce methane production.

The aim of the present study was to investigate the influence of *Hermetia illucens* larvae meal on the diversity and composition of methanogens in the caecum of laying hens (Hy-line Brown). A soybean protein source was partially substituted (25% and 50%) with *H. illucens* larvae meal in the hens’ diet and the caecal content was analysed using a 16S rRNA gene clone library to identify methanogenic archaea.

## Materials and methods

### Ethics statement

Animal handling and treatments were performed in accordance with requirements for the care and use of agriculture animals in research and all procedures were performed in concordance with the European legislation Directive 2010/63/EU on protecting animals for scientific purposes. Ethical approval for the experiment was granted by the Ethical Animal Care and Use Committee of the Department of Veterinary Medicine and Animal Production of the University of Napoli Federico II, Italy (prot. N. 2017/0017676). The trial took place in a private laying hen farm located in Sardinia (Italy).

### Animals and diet

A total of 30 animals were randomly selected from a cohort of 162 sixteen-weeks-old Hy-line Brown hens with an average weight of 1.41 kg ± 0.13. The hens were divided into 3 dietary treatment groups and housed for another 24 weeks in modified cages (800 cm^2^/hen, please add sizes of cages: cages (270 cm length × 54 cm depth × 45 cm height) under controlled temperature and humidity conditions. The hens were fed three isoproteic and isoenergetic diets. The control group (C) received a standard corn-soybean meal diet formulated to meet the nutritional requirements outlined in the Hy-line Brown commercial line management guide (Guide, 2016). In the HI25 and HI50 experimental groups, 25% and 50% of the soybean meal protein content, respectively, was replaced by the protein of partially defatted *Hermetia illucens* larvae meal purchased from the European company (HI, Hermetia Deutschland GmbH & Co. KG, Amtsgericht Potsdam, Germany). The diets were fed as flour and in order to avoid possible adverse effects on animal feed choice, all the ingredients were finely ground to achieve the same particle size and a homogeneous meal mixture. Feed and water were administered manually and feed intake was monitored daily. The dark:light cycle was 9:15 hours. The characteristics of the animals are given in Supplementary Table S1. The composition and chemical-nutritional characteristics of the diets were the same as in the work of Bovera et al. (2018) and are listed in Supplementary Tables S2 and S3.

### Samples collection

At 40 weeks of age, 9 hens were selected from each group and subsequently slaughtered. Following evisceration, the luminal contents of the caeca were collected in sterilised microcentrifuge tubes, frozen at -80°C and freeze-dried using the Heto powerdry LL3000 freeze dryer (Thermo Fisher Scientific, Wilmington, DE, USA). The samples were then transported to the Institute of Animal Physiology and Genetics of the Czech Academy of Sciences (Prague, Czech Republic) for further analysis.

### DNA extraction PCR amplification, clone library construction and sequencing

Genomic DNA was extracted from the freeze-dried caecal samples using PowerSoil DNA Kit (QIAGEN, Hilden, Germany) according to the manufacturer's instructions. The concentration and quality of the extracted DNAs were assessed using a NanoDrop 2000c UV-Vis spectrophotometer (Thermo Scientific, Wilmington, DE, USA) and stored at -20°C until until used. For further analysis, the 50 μL of DNA from each sample of animal of each treatment were mixed together, i.e. 9 × 50 μL of DNA for each group and these mixtures were used as template C, HI25 and HI50 for preparation three clone libraries. The PCR cloning products, approximately 1.2 kb in length, were generated using the methanogen specific primers (Met86F and Met1340R) (Wright and Pimm, 2003) as described by Murru et al. (2018). Briefly, thermal cycling conditions included a denaturation step for 4 min at 94°C, followed by 33 cycles of 1 min at 94°C, 30 s at 58°C, 45 s at 72°C and final elongation step at 72°C for 2 min. The TOPO® TA Cloning® Kit for Sequencing (Life Technologies, U.S.A) was used according to the manufacturer's instructions to generate archaeal 16S rRNA clone libraries for each group of hens. The positive *E. coli* clones were randomly selected, the recombinant plasmids were isolated using a GenElute™ HP Plasmid Miniprep Kit (Sigma-Aldrich, U.S.A.) and subjected to sequencing with the primer M13F (Seqme.eu, Dobříš, Czech Republic).

### Phylogenetic affiliation and statistical analysis

The sequences were edited using Chromas (version 2.6.6) and checked for chimeras (Wright et al., 2012). Geneious Prime was used to perform the alignment of the 16S rRNA gene sequences of methanogens and check their orientation in the plasmid. The BLAST tool on the NCBI website was utilized to identify the closest relatives of all sequenced clones, either the nearest validly named methanogens (cultured methanogens) or the nearest uncultured archaeal clone. MEGA11 software (Tamura et al., 2021) was used to generate the phylogenetic tree with 1,000 replicates of bootstrap using the UPGMA method. Evolutionary distances were calculated using the Maximum Composite Likelihood method (Tamura et al., 2004). Only the sequences with the same orientation were used for the phylogenetic analysis. The microbial diversity of caecal samples was assessed using the Shannon index (Shannon, 1948), in order to evaluate the species richness and evenness. The comparison among the groups was done with the Hutcheson t-test (Hutcheson, 1970). The sequences of control group, HI25 group and HI50 group were designated as MH1, MH3 and MH2, respectively. Sequence information was deposited in the GenBank database under the following accession numbers: PP631991-PP632098.

### Reference sequences used in of phylogenetic trees construction

The sequences of the 16S rRNA genes of the cultured methanogens *Methanimicrococcus blatticola* (AY196680), *Methanobacterium formicicum* (AF169245), *Methanobrevibacter olleyae* (NR043024), *Methanorbis furvi strain Ag1* (OQ442338), *Methanobrevibacter smithii* (AY196669), *Methanobrevibacter sp. NT7* (AJ009959), *Methanobrevibacter woesei strain CH389* (DQ445719), *Methanobrevibacter woesei strain GS* (NR044788), *Methanococcus vannielii* (AY196675), *Methanocorpusculum bavaricum* (NR042787), *Methanocorpusculum labreanum* (NR074173), *Methanofollis liminatans* (Y16429), *Methanogenium thermophilum* (M59129), *Methanomassiliicoccus luminyensis* (NR118098), *Methanomicrobium mobile* (AY196679), *Methanospirillum hungatei* (AY196683), *Methanosarcina barkeri* (AY196682), *Methanomassiliicoccaceae archaeon DOK* (CP047880) complemented with the highest similarity sequences of uncultured archeons *Uncultured Methanocorpusculum sp. clone Ag562* (OP852044), *Uncultured archaeon clone: PMET30* (AB739382), *Methanogenic archaeon CH1270* (DQ445723) assessed by the online blast search were used as the references in the phylogenetic trees. The *Aquifex pyrophilus* (NR029172) was used as the outgroup for rooting the trees.

## Results

### Diversity of archaeal communities

**Fig. 1:**
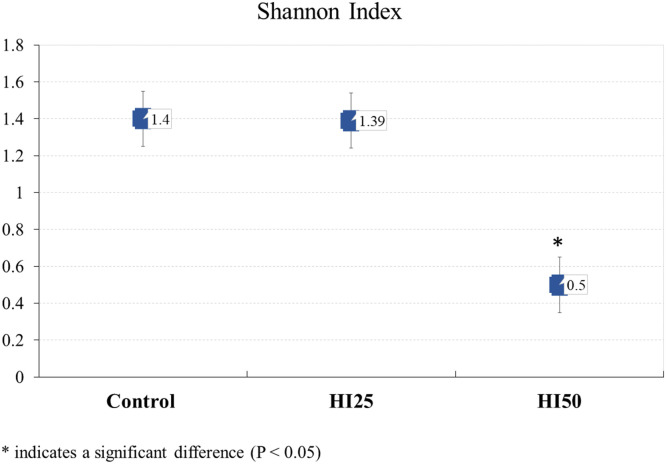
Comparison of Shannon diversity indices of the caecal methanogen communities of three groups of hens fed different diets. The Hutcheson t-test was used for the sample comparison.

A total of 108 clones from 3 archaeal 16S rRNA gene cloning libraries from the caeca of hens fed different diets were analyzed. The clones from control group (35 positive clones), HI25 group (44 positive clones) and HI50 group (29 positive clones) were sequenced, compared with the GenBank data and evaluated for microbial diversity. The microbial diversity of the caecal samples was assessed using the Shannon index (Shannon, 1948), in order to evaluate the species richness and evenness. Statistical comparisons among the groups were performed using the Hutcheson t-test (Hutcheson, 1970), with significance set at P < 0.05. The analysis revealed lower richness and evenness in the HI50 group (Table 1 and Fig. 1). The Shannon index showed a significant difference between the control group and the HI50 group (P = 2.4 × 10^-5^) and also between the HI25 and HI50 groups (P = 2.5 × 10^-5^) as summarized in Supplementary Table S2.

**Table 1:** Diversity analysis of the methanogenic 16S rRNA gene clone libraries generated from the caecal content of laying hens fed different diets.

Diet	Number of clones	Richness	Shannon index
**Control**	35	5	1.40
**HI25**	44	5	1.39
**HI50**	29	3	0.50

### Phylogenetic affiliation of the 16S rRNA gene sequences

All 16S rRNA sequences generated from the caecal samples were subjected to BLAST search against GenBank data. More than 77% of the sequences were affiliated to cultured methanogens belonging to 3 orders: Methanomicrobiales, Methanomassiliicoccales and Methanobacteriales. The sequenced clones with their nearest valid taxa, percentage identity, query cover and accession number are listed in the Table S3. Regardless of the hen's diet, the major order was Methanobacteriales (C: 37.1%; HI25: 43.2%; HI50: 86.2%), followed by Methanomassiliicoccales in the control group, while in the HI25 and HI50 groups Methanomicrobiales was the second most prevalent order. Methanomassiliicoccales was not found in HI50 group.

**Fig. 2:**
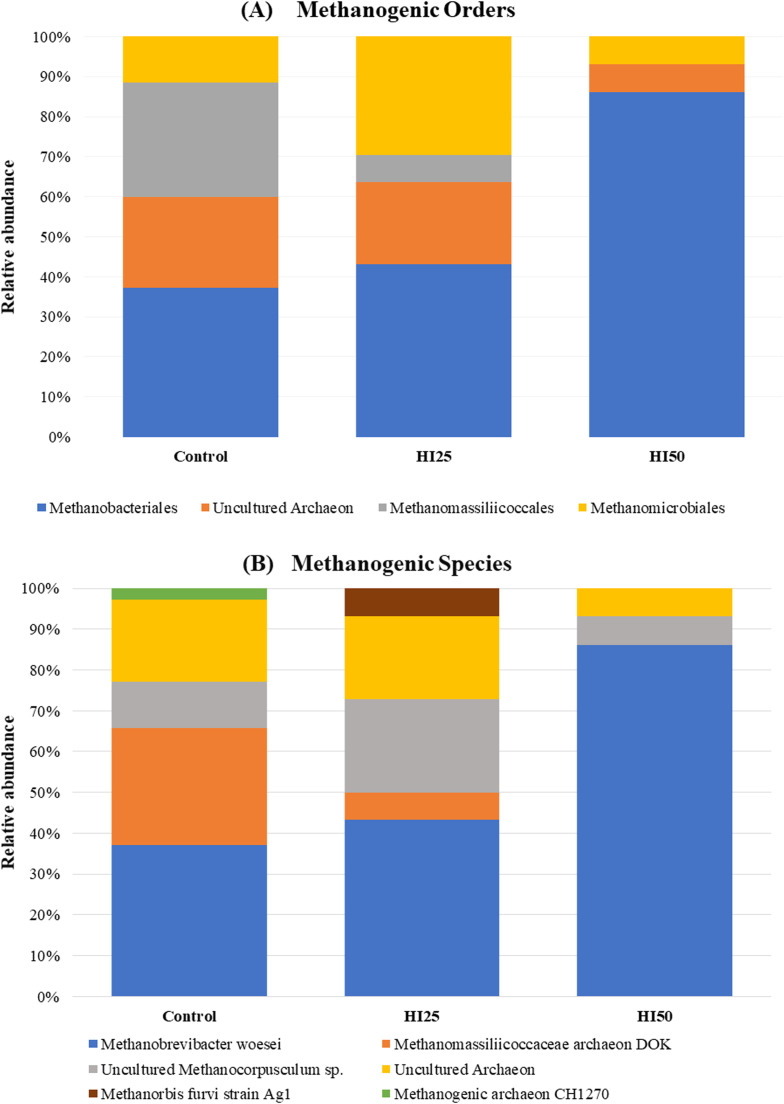
Relative abundances of caecal methanogenic archaea at order (A) and species (B) levels in three groups of hens fed different diets.

At the species level, *Methanobrevibacter woesei* was the most prevalent archaeon across all three groups, however considerable variations in the relative abundances were observed within the archaeal community among the different diets. In the control group, the second most dominant species was *Methanomassiliicoccaceae archaeon DOK* followed by *uncultured Methanocorpusculum sp*. In addition, *Methanogenic archaeon CH1270* was only observed in the control group albeit in low relative abundance. In the HI25 group, the second most dominant species was *uncultured Methanocorpusculum sp.*, followed by *Methanomassiliicoccaceae archaeon DOK. Methanorbis furvi strain Ag1* was found exclusively in the HI25 group although in limited relative abundance. Species diversity was significantly lower in the HI50 group, with the majority of sequences related to *Methanobrevibacter woesei* (86.2%), followed by a small amount of *uncultured Methanocorpusculum sp*. The percentage relative abundances at the order and species level are shown in Fig. 2 and listed in Supplementary Table S4.

**Fig. 3:**
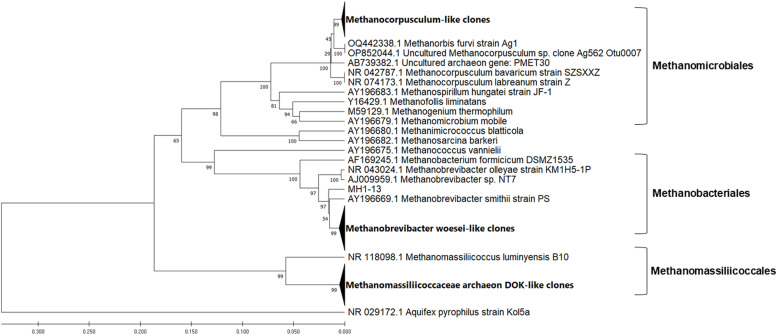
Phylogenetic relationships of the archaeal clones derived from 16S rRNA gene sequences of caecal sample of hens fed the standard corn-soybean meal diet (control group). The evolutionary history was inferred using the UPGMA method with bootstrap values from 1000 replications. The evolutionary distances were calculated using the Maximum Composite Likelihood method. The analysis involved 57 nucleotide sequences and was conducted in MEGA11.

**Fig. 4:**
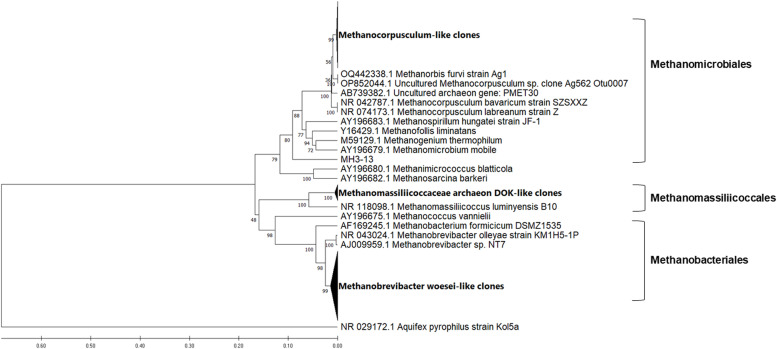
Phylogenetic relationships of the archaeal clones derived from 16S rRNA gene sequences of caecal sample of hens fed a diet with 25% replacement of the soybean meal protein by the protein from *H. illucens* (HI25 group). The evolutionary history was inferred using the UPGMA method with bootstrap values from 1000 replications. The evolutionary distances were calculated using the Maximum Composite Likelihood method. The analysis involved 66 nucleotide sequences and was conducted in MEGA11.

**Fig. 5:**
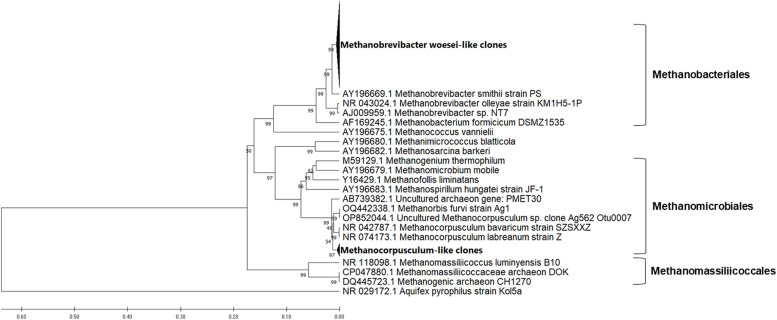
Phylogenetic relationships of the archaeal clones derived from 16S rRNA gene sequences of caecal sample of hens fed a diet with 50% replacement of the soybean meal protein by the protein from *H. illucens* (HI50 group). The evolutionary history was inferred using the UPGMA method with bootstrap values from 1000 replications. The evolutionary distances were calculated using the Maximum Composite Likelihood method. The analysis involved 51 nucleotide sequences and was conducted in MEGA11.

The phylogenetic analyses of the sequences generated from caeca of hens fed three different diets are shown in Figs. 3, Fig. 4, Fig. 5. The maximum composite likelihood analysis of the cloned sequences from control group of hens revealed three distinct clusters (Fig. ​3). The cluster of Methanobacteriales was composed of 12 sequences with high similarity to *Methanobrevibacter woesei* (NR044788, DQ445719), forming sister subcluster to *M. smithii* (AY196669). The cluster of Methanomassiliicoccales contained the subcluster of our sequences (11 sequences) with high similarity to Methanomassiliicoccaceae archeon DOK (CP047880) and uncultured methanogenic archeon CH1270 (DQ445723), forming the sister cluster to *Methanomassiliicoccus luminyensis* (NR118098). The cluster of Methanomicrobiales contained the subcluster of our sequences (11 sequences) with high similarity to uncultured *Methanocorpusculum* OP852044. All these sequences formed a sister cluster to cultured *Methanocorpusculum bavaricum* (NR042787) and *Methanocorpusculum labreanum* (NR074173).

Three distinct clusters were also generated by analysis of the cloned sequences from HI25 group of hens (Fig. 4). Only three sequences grouped with Methanomassiliicoccales forming the separate subcluster with high similarity to Methanomassiliicoccaceae archeon DOK (CP047880) and uncultured methanogenic archeon CH1270 (DQ445723). The majority of sequences (21 sequences) grouped in separate subcluster in Methanomicrobiales and formed a sister cluster to *Methanocorpusculum* strains. Only one sequence (MH3_13) clustered separately within the Methanomicrobiales order. The rest of sequences (19 sequences) formed consistent cluster in Methanobacteriales with high similarity to *Methanobrevibacter woesei* (NR044788, DQ445719).

The methanogenic sequences generated from caecal sample of HI50 group of hens were divided into two clusters. The majority of them (25 sequences) formed well supported subcluster in Methanobacteriales with high similarity to *Methanobrevibacter woesei* (NR044788, DQ445719), while only 4 sequences clustered separately in Methanomicrobiales, forming sister cluster to *Methanocorpusculum* strains.

## Discussion

The gastrointestinal microbiome plays an essential role in the health of poultry, influencing digestion, productivity, immune system and overall animal welfare (Clavijo and Flórez, 2018). The caecum has been identified as a key region where the highest microbial diversity is found (Oakley et al., 2014). The microbiota in this intestinal segment consists mainly of bacteria. Methanogens are represented to a much lesser extent representing approximately 1% (Qu et al., 2008). However, methanogenic archaea play the essential role in maintaining the hydrogen balance in the GIT (Cisek et al., 2023), because the majority of the methanogens are hydrogenotrophic using H_2_ to reduce CO_2_ into methane (Liu and Whitman, 2008). Hydrogen depletion optimizes fermentation and, in metabolic synergy with beneficial microorganisms, can increase the host animal's capacity to obtain additional energy from the diet (Saengkerdsub and Ricke, 2014). On the other hand, methane production has negative impacts, because CH_4_ is the second most important greenhouse gas contributing to global warming (after carbon dioxide) and represents a loss of ingested feed-derived energy for the animal (2- 12% of gross energy intake). Even if the contribution of poultry production (including eggs) to climate changes is the lowest (9.8%) in livestock sector (Tetteh et al., 2022), a study of the methanogens in non-ruminants is very important with respect to the enormous increase in poultry production (by 807.8% in last thirty years) (World,) and various methane mitigation strategies, including dietary strategies and use of functional supplements (Misiukiewicz et al., 2021).

Our results showed that the replacement of corn-soybean meal with *Hermetia illucens* larvae meal in the diet of laying hens was reflected in the altered diversity of methanogens, depending on the percentage of insect meal included in the diet. Twenty-five percent of *H. illucens* larvae meal in the diet influenced the diversity, but no statistically significant difference was observed (HI25 group), while fifty percent significantly suppressed methanogenic richness and evenness (HI50 group). This finding is in agreement with the study of Biasato et al. (2020) who revealed a lower Shannon index for bacterial diversity in broilers fed 15% *H. illucens* meal. However, several other studies have found no significant difference in microbial diversity (Kawasaki et al., 2019; Vilela et al., 2023), while others revealed an increase in diversity (Yan et al., 2023; Huang et al., 2024). These differences can be related to the variation in the level of inclusion and the nutritional quality of the insect meal. The distinct response of methanogens and bacteria to the inclusion of *H. illucens* larvae meal in the diet could also contribute to these differences. Reduced diversity is considered a negative effect. Gut microbiome with low diversity is related to the poor microbial stability and animal´s health status, as functions of the microbiota are associated with nutrients production, protection against pathogens and maturation of the host immune system (Aruwa et al., 2021).

Based on our preliminary results, the methanogenic composition in hens’ caecal samples is dominated by sequences with high similarity to *M. woesei* species (order Methanobacteriales) regardless of the type of diet. This finding is in agreement with Saengkerdsub et al. (2007), who identified this archeon as the predominant methanogen in chicken caeca. In our study, this species was prevalent especially in HI50 group, which may indicate a supportive effect of insect meal on this mathanogen. *M. woesei* produces methane from H_2_ and CO_2_ (Miller and Lin, 2002) and formate is used to a small extent. There is surprisingly low amount of information on this species and published data could suggest that this methanogen is linked to the digestive tract of poultry (Miller et al., 1986; Saengkerdsub et al., 2007). The *Methanobrevibacter* is otherwise widespread genus and belongs to the most predominant methanogens in human and animals (Saengkerdsub and Ricke, 2014), where is however represented by other species, such as *M. ruminantium, M. smithii* or *M. gottschalkii* (Aryee et al., 2023).

The second most prevalent type of sequences in the control group showed a high similarity to the *Methanomassiliicoccaceae archaeon DOK* (order Methanomassiliicoccales), whereas this archaeon was repressed in the HI25 group and completely absent in the HI50 group. Methanomassilliicocales is recently defined order within the Euryarchaeota phylum and only limited genomic and functional data are available (Xie et al., 2024). This order consists of one family with considerable variation at the genus level. The importance of Methanomassiliicoccales insists in the possession of genes involved in the anaerobic B_12_ biosynthesis pathway, which plays a crucial role in their metabolic processes. Methanomassiliicoccales are distinguished from other methanogenic orders by the presence of diverse clades of vitamin transporter BtuC proteins, which property is probably associated with competitive advantage in effective handling of B12 (Xie et al., 2024). Another interesting point results from studies of ruminants, which differ in the amount of methane emissions, indicating increased expression of selected Methanomassiliicoccales genera in animals with low methane production.

Sequences similar to ucultured *Methanocorpusculum* species (order Methanomicrobiales) were present in all three groups of partridges, but a higher abundance was detected in HI25 group. *Methanorbis furvi strain Ag1* was also found exclusively in the HI25 group although in limited relative abundance. *Methanocorpusculum* has long been considered as environmental methanogen inhabiting freshwater, bogs and soils (Volmer et al., 2023), but now is well-known to be present in the gut of various animal hosts, including termites (Ohkuma et al., 1995), reptiles (Thomas et al., 2022), hindgut animals (Luo et al., 2013; Murru et al., 2018; Guindo et al., 2021) and foregut animals (Li et al., 2019). *Methanocorpusculum* in poultry has only recently been described in faecal samples of hens (Gilroy et al., 2021) and broiler chicken (Wang et al., 2024). On the other hand, Miller et al. (Miller et al., 1986) did not isolate any *Methanocorpusculum* from samples of the chicken and turkey and indicated *Methanogenium* to be a major component of feces of these birds. However, results of the cultivation effort are not comparable with the results based on the molecular biological approach due to the strong dependence on the type of nutrient broth. *Methanocorpusculum*, like all members of the order Methanomicrobiales, is obligate hydrogenotroph producing methane from H_2_ and CO_2_, some species can use also formate (Welte and Deppenmeier, 2014). *Methanocorpusculum* remains inadequately characterized due to the low amount of cultivated species and Methanomicrobiales is the least explored taxon among methanogens (Anderson et al., 2009). The pyrosequencing analysis of Qu et al. (Qu et al., 2008) may indicate that this order plays an important role in chicken caeca as it can represent 45-47% of the archaeal composition. However, these results should be treated with caution due to the small number of samples analyzed.

Our results indicate a notable influence of HI larvae meal on the archaeal composition of caecum of hens showing shifts in the belonging of sequences to different species of methanogens, which may be hypothetically related to the amount of produced methane. Several *in vitro* studies of the effect of a substitution of soybean meal with various insect meals in ruminant diets showed a reduction in methane production (Jayanegara et al., 2017a; b; Ahmed et al., 2021; Phesatcha et al., 2022). These interesting results suggest that insect meal could be a challenging tool to influence methane emissions. Due to the large and constantly increasing production of broiler chickens and laying hens, this aspect should be taken into account in studies of the impact of insect meal on the diversity of intestinal methanogens, methane production of poultry and possible methane mitigation.

One of the key strengths of this study is its novelty, as it provides the first insights into how insect meal influences methanogenic archaea in poultry. The use of molecular methods, such as 16S rRNA gene clone libraries, enabled a detailed analysis of the methanogenic community and provided valuable data on the shifts in archaeal diversity and composition. However, a limitation of this study is that methane emissions were not directly measured, which would have provided a more comprehensive understanding of the relationship between methanogen diversity and methane production. Furthermore, the study only focused on short-term dietary interventions and the long-term effects of insect meal on methanogen populations remain unknown. Future research should address these limitations by including direct methane emission measurements and long-term dietary trials to better understand the functional implications of these changes in methanogenic communities.

## Conclusion

To the best of our knowledge, this is the first study evaluating the effect of *H. illucens* larvae meal dietary inclusion on the caecal methanogenic diversity of laying hens. The results indicate that used insect meal may have an important impact on the caecal archaeal composition, leading to changes in the relative abundance of some taxa and a decrease in richness and evenness when 50% of the soybean meal protein of the diet is substituted with insect meal protein. Due to the paucity of literature data, it is difficult to evaluate whether the induced changes are positive or negative. Future studies should investigate the long-term effects of insect meal on methanogen populations and their functional roles in methane production. Additionally, direct measurements of methane emissions in response to insect meal intake would provide valuable insights into the relationship between methanogen diversity and methane production and help determine the optimal levels of insect meal inclusion which support gut health without negatively impacting methanogenic diversity. These findings could contribute to the development of sustainable dietary strategies for poultry production, balancing the benefits of insect meal with the need to mitigate greenhouse gas emissions.

## Author contributions

Conceptualization, K.O.F., F.B. and G.M.; methodology, K.O.F., J.M. and T.M.M; formal analysis and investigation, T.M.M.; resources, F.B and G.M.; data curation, T.M.M.; writing—original draft preparation, T.M.M.; writing—review and editing, K.O.F., F.B. and G.M. All authors have read and agreed to the published version of the manuscript.

## Data availability statement

The data presented in this study are openly available in the GenBank under the accession numbers PP631991-PP632098.

## Disclosures

The authors declare the following financial interests/personal relationships which may be considered as potential competing interests:

Giuseppe Moniello reports financial support was provided by Sardegna Foundation. If there are other authors, they declare that they have no known competing financial interests or personal relationships that could have appeared to influence the work reported in this paper.

## Appendix Supplementary materials

Image, application 1
