# Supplementary material for: The inclusion of insect meal from Hermetia illucens larvae in the diet of laying hens (Hy-line Brown) affects the caecal diversity of methanogenic archaea
Source: Poult Sci. 2025 Mar 15;104(5):105037. doi: 10.1016/j.psj.2025.105037 (PMC11987624; doi:10.1016/j.psj.2025.105037)
Supplement: Supplementary file 1 [file mmc1.docx]

**Supplementary Material**

**Modulation of caecal methanogenic archaea by dietary insect meal from *Hermetia illucens* larvae in laying hens (Hy-line Brown)**

Tiziana Maria Mahayri ^*, †^, Jakub Mrázek ^*^, Fulvia Bovera ^‡^, Giovanni Piccolo ^‡^, Giovanni Andrea Murgia ^§^, Giuseppe Moniello ^†^, Kateřina Olša Fliegerová ^*^

^*^ Laboratory of Anaerobic Microbiology, Institute of Animal Physiology and Genetics, Czech Academy of Science, 14220 Prague, Czech Republic

^†^ Department of Veterinary Medicine, University of Sassari, 07100 Sassari, Italy

^‡^  Department of Veterinary Medicine and Animal Production, University of Napoli Federico II, via F. Delpino, 1, 80137 Napoli, Italy

^§^ External Collaborator Veterinary Practitioner, 07100 Sassari, Italy

Corresponding author: moniello@uniss.it; Tel.: +39 079 229443

Section: Microbiology and Food Safety

**Table S1.** Animals’ characteristics and samples weight for DNA extraction.

| **Samples** | **Diet** | **Age** | **Weight for DNA extraction** |
| --- | --- | --- | --- |
| H 1_2 | Control | 40 weeks | 80mg |
| H 1_3 | Control | 40 weeks | 79mg |
| H 1_4 | Control | 40 weeks | 79mg |
| H 1_5 | Control | 40 weeks | 83mg |
| H 1_6 | Control | 40 weeks | 78mg |
| H 1_7 | Control | 40 weeks | 80mg |
| H 1_8 | Control | 40 weeks | 79mg |
| H 1_9 | Control | 40 weeks | 81mg |
| H 1_10 | Control | 40 weeks | 80mg |
| H 2_1 | H50 | 40 weeks | 93mg |
| H 2_2 | H50 | 40 weeks | 136mg |
| H 2_3 | H50 | 40 weeks | 101mg |
| H 2_5 | H50 | 40 weeks | 112mg |
| H 2_6 | H50 | 40 weeks | 145mg |
| H 2_7 | H50 | 40 weeks | 118mg |
| H 2_8 | H50 | 40 weeks | 140mg |
| H 2_9 | H50 | 40 weeks | 118mg |
| H2_10 | H50 | 40 weeks | 105mg |
| H 3_1 | H25 | 40 weeks | 90mg |
| H 3_2 | H25 | 40 weeks | 81mg |
| H 3_3 | H25 | 40 weeks | 102mg |
| H 3_4 | H25 | 40 weeks | 88mg |
| H 3_5 | H25 | 40 weeks | 99mg |
| H 3_6 | H25 | 40 weeks | 101mg |
| H 3_7 | H25 | 40 weeks | 87mg |
| H 3_8 | H25 | 40 weeks | 85mg |
| H 3_9 | H25 | 40 weeks | 80mg |

**Table S2.** Proximate composition, mineral and essential amino acid composition (% as fed) of the *Hermetia illucens* larvae meal and soybean meal.

|  | ***Hermetia illucens* Larvae meal** | **Soybean meal** |
| --- | --- | --- |
| **Proximate composition** | | |
| Dry matter | 92.7 | 90.0 |
| Crude protein | 55.6 | 43.4 |
| Ether extract | 8.34 | 1.1 |
| ADF | 11.5 | 5.9 |
| ADF-linked protein | 4.86 | 1.78 |
| Ash | 7.8 | 6.0 |
| **Mineral composition** | | |
| Ca^a^ | 6.47 | 2.83 |
| Total P^a^ | 0.90 | 0.57 |
| Na^a^ | 0.12 | 0.16 |
| **Essential amino acid composition** | | |
| Lysine^a^ | 4.12 | 2.92 |
| Methionine^a^ | 1.09 | 0.61 |
| Methionine + Cystine^a^ | 1.32 | 1.33 |
| Isoleucine^a^ | 2.97 | 2.30 |
| Tryptophan^a^ | 0.30 | 0.73 |
| Valine^a^ | 5.02 | 2.11 |
| Threonine^a^ | 2.32 | 1.74 |

^a^ Obtained by manufacturing company.

**Table S3.** Ingredients and chemical-nutritional characteristics of diets.

|  | **C** | **HI25** | **HI50** |
| --- | --- | --- | --- |
| **Ingredients, g/kg** | | | |
| Maize grain | 606 | 598 | 631 |
| Soybean meal | 265 | 200 | 95 |
| Insect meal | – | 73 | 146 |
| CaCO_3_ grains | 80 | 80 | 80 |
| Vegetable oil | 10 | 10 | – |
| MinVit^c^ | 10 | 10 | 10 |
| Methionine | 2.5 | 2.0 | 2.0 |
| Monocalcium phosphate | 5 | 5 | 5 |
| Celite | 20 | 20 | 20 |
| Salt | 2 | 2 | 2 |
| **Chemical-nutritional characteristics** | | | |
| Dry matter,^a^ % | 91.53 | 91.39 | 91.62 |
| Crude protein,^a^ % | 16.45 | 16.32 | 17.03 |
| Ether extract,^a^ % | 3.37 | 3.51 | 3.86 |
| NDF,^a^ % | 10.38 | 11.29 | 12.49 |
| ADF,^a^ % | 5.85 | 5.90 | 5.67 |
| ADL,^a^ % | 2.67 | 2.94 | 2.29 |
| Lysine,^b^ % | 0.90 | 0.97 | 1.00 |
| Methionine,^b^ % | 0.53 | 0.54 | 0.54 |
| Metabolizable Energy,^b^ kcal/kg | 2832.3 | 2845.2 | 2842.2 |

C: soybean meal based diet; HI25: diet including Hermetia illucens as 25% of replacement of the soybean meal protein; HI50: diet including *Hermetia illucens* as 50% of replacement of the soybean meal protein;

a Determined according to AOAC (2005)

b Calculated according to NRC (1994)

c Provided per kilogram: vitamin A (retinyl acetate) 20,000 IU, vitamin D3 (cholecalciferol) 6000 IU, vitamin E (dl-α-tocopheryl acetate) 80 IU, vitamin B1(thiamine monophosphate) 3 mg, vitamin B2 (riboflavin) 12 mg, vitamin B6 (pyridoxine hydrochloride) 8 mg, vitamin B12 (cyanocobalamin) 0.04 mg, vitamin K3 (menadione) 4.8 mg, vitamin H (d biotin) 0.2 mg, vitamin PP (nicotinic acid) 48 mg, folic acid 2 mg, calcium pantothenate 20 mg, manganous oxide 200 mg, ferrous carbonate 80 mg, cupric sulphate pentahydrate 20 mg, zinc oxide 120 mg, basic carbonate monohydrate 0.4 mg, anhydrous calcium iodate 2 mg, sodium selenite 0.4 mg, choline chloride 800 mg, 4–6-phitase 1800 FYT, D.L. methionine 2600 mg, canthaxanthin 8 mg.

**Table S4.** Effects of diet on diversity indices of archaeal community in hens’ caecum (*p*-values).

| **Diet** | **Shannon Index**  *P*-value |
| --- | --- |
| Control vs HI25 | 0.9 |
| Control vs HI50 | 2.4 x 10^-5^* |
| HI25 vs HI50 | 2.5 x 10^-5^* |

* Significant difference (p < 0.05)

**Table S5.** Sequenced clones with their nearest valid taxa, percentage identity, query cover and accession number.

| **Clones** | **Taxonomy** | **Percentage Identity** | **Query Cover** | **Accession** |
| --- | --- | --- | --- | --- |
| MH1_5 | Uncultured archaeon | 99.48% | 85% | GU391231.1 |
| MH1_6 | Uncultured Methanocorpusculum sp. | 97.59% | 95% | OP852044.1 |
| MH1_7 | Methanomassiliicoccaceae archaeon DOK | 99.08% | 100% | CP047880.1 |
| MH1_8 | Methanomassiliicoccaceae archaeon DOK | 98.53% | 100% | CP047880.1 |
| MH1_9 | Uncultured Methanocorpusculum sp. | 97.40% | 99% | OP852044.1 |
| MH1_10 | Uncultured archaeon | 99.58% | 89% | GU391231.1 |
| MH1_11 | Methanomassiliicoccaceae archaeon DOK | 99.07% | 100% | CP047880.1 |
| MH1_12 | Methanobrevibacter woesei | 98.34% | 100% | NR_044788.1 |
| MH1_13 | Methanobrevibacter woesei | 96.51% | 99% | NR_044788.1 |
| MH1_14 | Methanobrevibacter woesei | 99.44% | 99% | NR_044788.1 |
| MH1_16 | Uncultured archaeon | 96.64% | 99% | AB739382.1 |
| MH1_17 | Methanomassiliicoccaceae archaeon DOK | 99.72% | 100% | CP047880.1 |
| MH1_18 | Uncultured archaeon | 96.54% | 99% | AB739382.1 |
| MH1_19 | Uncultured archaeon | 96.64% | 99% | AB739382.1 |
| MH1_20 | Methanobrevibacter woesei | 98.89% | 100% | NR_044788.1 |
| MH1_21 | Uncultured archaeon | 96.45% | 99% | AB739382.1 |
| MH1_23 | Uncultured Methanocorpusculum sp. | 97.67% | 100% | OP852044.1 |
| MH1_24 | Methanobrevibacter woesei | 99.53% | 100% | NR_044788.1 |
| MH1_25 | Methanomassiliicoccaceae archaeon DOK | 99.81% | 100% | CP047880.1 |
| MH1_27 | Methanogenic archaeon CH1270 | 99.53% | 100% | DQ445723.1 |
| MH1_28 | Methanobrevibacter woesei | 98.07% | 100% | NR_044788.1 |
| MH1_29 | Methanobrevibacter woesei | 99.16% | 100% | NR_044788.1 |
| MH1_30 | Methanobrevibacter woesei | 99.16% | 100% | NR_044788.1 |
| MH1_32 | Methanomassiliicoccaceae archaeon DOK | 99.35% | 100% | CP047880.1 |
| MH1_33 | Uncultured Methanocorpusculum sp. | 97.76% | 100% | OP852044.1 |
| MH1_35 | Uncultured archaeon | 96.64% | 99% | AB739382.1 |
| MH1_36 | Methanobrevibacter woesei | 96.10% | 100% | NR_044788.1 |
| MH1_37 | Methanomassiliicoccaceae archaeon DOK | 99.35% | 100% | CP047880.1 |
| MH1_38 | Methanobrevibacter woesei | 99.35% | 99% | NR_044788.1 |
| MH1_40 | Methanomassiliicoccaceae archaeon DOK | 99.81% | 100% | CP047880.1 |
| MH1_41 | Methanomassiliicoccaceae archaeon DOK | 99.81% | 100% | CP047880.1 |
| MH1_42 | Methanomassiliicoccaceae archaeon DOK | 99.72% | 100% | CP047880.1 |
| MH1_44 | Methanobrevibacter woesei | 98.61% | 99% | NR_044788.1 |
| MH1_45 | Methanobrevibacter woesei | 99.16% | 100% | NR_044788.1 |
| MH1_47 | Methanobrevibacter woesei | 99.07% | 99% | NR_044788.1 |
| MH2_2 | Methanobrevibacter woesei | 99.53% | 100% | NR_044788.1 |
| MH2_3 | Uncultured Methanocorpusculum sp. | 97.85% | 95% | OP852044.1 |
| MH2_6 | Methanobrevibacter woesei | 99.44% | 95% | NR_044788.1 |
| MH2_7 | Methanobrevibacter woesei | 99.16% | 95% | NR_044788.1 |
| MH2_8 | Methanobrevibacter woesei | 99.07% | 95% | NR_044788.1 |
| MH2_11 | Methanobrevibacter woesei | 98.97% | 95% | NR_044788.1 |
| MH2_12 | Methanobrevibacter woesei | 99.35% | 95% | NR_044788.1 |
| MH2_13 | Methanobrevibacter woesei | 99.44% | 95% | NR_044788.1 |
| MH2_15 | Methanobrevibacter woesei | 99.16% | 95% | NR_044788.1 |
| MH2_20 | Methanobrevibacter woesei | 99.44% | 95% | NR_044788.1 |
| MH2_21 | Methanobrevibacter woesei | 99.44% | 95% | NR_044788.1 |
| MH2_22 | Methanobrevibacter woesei | 99.53% | 95% | NR_044788.1 |
| MH2_23 | Methanobrevibacter woesei | 99.44% | 95% | NR_044788.1 |
| MH2_24 | Methanobrevibacter woesei | 99.44% | 95% | NR_044788.1 |
| MH2_25 | Methanobrevibacter woesei | 99.54% | 97% | NR_044788.1 |
| MH2_26 | Methanobrevibacter woesei | 99.26% | 96% | NR_044788.1 |
| MH2_29 | Methanobrevibacter woesei | 99.44% | 95% | NR_044788.1 |
| MH2_30 | Methanobrevibacter woesei | 99.35% | 95% | NR_044788.1 |
| MH2_31 | Methanobrevibacter woesei | 99.16% | 95% | NR_044788.1 |
| MH2_32 | Methanobrevibacter woesei | 99.35% | 95% | NR_044788.1 |
| MH2_33 | Methanobrevibacter woesei | 99.26% | 95% | NR_044788.1 |
| MH2_37 | Methanobrevibacter woesei | 99.26% | 95% | NR_044788.1 |
| MH2_41 | Uncultured archaeon | 99.38% | 87% | GU391231.1 |
| MH2_42 | Methanobrevibacter woesei | 99.35% | 96% | NR_044788.1 |
| MH2_45 | Methanobrevibacter woesei | 99.35% | 95% | NR_044788.1 |
| MH2_46 | Methanobrevibacter woesei | 99.44% | 95% | NR_044788.1 |
| MH2_47 | Methanobrevibacter woesei | 99.44% | 96% | NR_044788.1 |
| MH2_48 | Uncultured Methanocorpusculum sp. | 97.67% | 95% | OP852044.1 |
| MH2_50 | Uncultured archaeon | 96.55% | 95% | AB541788.1 |
| MH3_1 | Uncultured Methanocorpusculum sp. | 98.04% | 95% | OP852044.1 |
| MH3_2 | Uncultured Methanocorpusculum sp. | 98.04% | 95% | OP852044.1 |
| MH3_3 | Methanomassiliicoccaceae archaeon DOK | 99.72% | 95% | CP047880.1 |
| MH3_4 | Uncultured Methanocorpusculum sp. | 98.04% | 95% | OP852044.1 |
| MH3_5 | Uncultured archaeon | 96.64% | 95% | AB541788.1 |
| MH3_6 | Methanobrevibacter woesei | 99.16% | 95% | NR_044788.1 |
| MH3_7 | Methanobrevibacter woesei | 99.25% | 95% | NR_044788.1 |
| MH3_8 | Methanobrevibacter woesei | 99.16% | 95% | NR_044788.1 |
| MH3_9 | Methanorbis furvi strain Ag1 | 96.55% | 95% | OQ442338.1 |
| MH3_10 | Methanorbis furvi strain Ag1 | 96.74% | 95% | OQ442338.1 |
| MH3_11 | Methanobrevibacter woesei | 99.35% | 95% | NR_044788.1 |
| MH3_12 | Methanobrevibacter woesei | 99.44% | 95% | NR_044788.1 |
| MH3_13 | Uncultured archaeon | 90.39% | 85% | GU391231.1 |
| MH3_15 | Methanobrevibacter woesei | 99.44% | 95% | NR_044788.1 |
| MH3_16 | Methanobrevibacter woesei | 99.53% | 95% | NR_044788.1 |
| MH3_17 | Uncultured archaeon | 96.64% | 95% | AB739382.1 |
| MH3_18 | Methanobrevibacter woesei | 99.53% | 95% | NR_044788.1 |
| MH3_19 | Uncultured archaeon | 96.73% | 95% | AB739382.1 |
| MH3_20 | Methanobrevibacter woesei | 99.44% | 95% | NR_044788.1 |
| MH3_21 | Uncultured archaeon | 96.83% | 95% | AB541788.1 |
| MH3_22 | Uncultured Methanocorpusculum sp. | 97.85% | 95% | OP852044.1 |
| MH3_23 | Methanobrevibacter woesei | 99.35% | 95% | NR_044788.1 |
| MH3_24 | Uncultured archaeon | 96.29% | 95% | AB541788.1 |
| MH3_26 | Uncultured Methanocorpusculum sp. | 97.95% | 95% | OP852044.1 |
| MH3_27 | Methanobrevibacter woesei | 99.29% | 95% | NR_044788.1 |
| MH3_28 | Methanobrevibacter woesei | 99.44% | 95% | NR_044788.1 |
| MH3_29 | Methanobrevibacter woesei | 99.44% | 95% | NR_044788.1 |
| MH3_30 | Uncultured Methanocorpusculum sp. | 97.86% | 95% | OP852044.1 |
| MH3_31 | Uncultured Methanocorpusculum sp. | 97.95% | 95% | OP852044.1 |
| MH3_32 | Methanomassiliicoccaceae archaeon DOK | 98.86% | 95% | CP047880.1 |
| MH3_34 | Methanomassiliicoccaceae archaeon DOK | 98.97% | 95% | CP047880.1 |
| MH3_35 | Methanorbis furvi strain Ag1 | 96.65% | 95% | OQ442338.1 |
| MH3_37 | Methanobrevibacter woesei | 98.60% | 95% | NR_044788.1 |
| MH3_38 | Uncultured Methanocorpusculum sp. | 97.95% | 95% | OP852044.1 |
| MH3_39 | Uncultured Methanocorpusculum sp. | 97.86% | 95% | OP852044.1 |
| MH3_41 | Uncultured archaeon | 96.73% | 95% | AB739382.1 |
| MH3_42 | Methanobrevibacter woesei | 99.53% | 95% | NR_044788.1 |
| MH3_43 | Methanobrevibacter woesei | 99.26% | 96% | NR_044788.1 |
| MH3_44 | Methanobrevibacter woesei | 99.16% | 95% | NR_044788.1 |
| MH3_46 | Uncultured Methanocorpusculum sp. | 98.04% | 95% | OP852044.1 |
| MH3_47 | Methanobrevibacter woesei | 99.35% | 95% | NR_044788.1 |
| MH3_48 | Methanobrevibacter woesei | 99.26% | 95% | NR_044788.1 |
| MH3_49 | Uncultured archaeon | 96.36% | 95% | AB739382.1 |
| MH3_50 | Uncultured archaeon | 96.64% | 95% | AB541788.1 |

**Table S6.** Percentage relative abundances of archaeal taxa at order and species level in the caecum of hens.

| **Taxonomic levels** | **Control** | **HI25** | **HI50** |
| --- | --- | --- | --- |
| **Orders** |  |  |  |
| Methanomicrobiales | 11.4% | 29.5% | 6.9% |
| Methanomassiliicoccales | 28.6% | 6.8% | 0 |
| Methanobacteriales | 37.1% | 43.2% | 86.2% |
| Uncultured Archaeon | 22.8% | 20.4% | 6.9% |
| **Species** |  |  |  |
| [*Uncultured Methanocorpusculum sp.*](https://www.ncbi.nlm.nih.gov/Taxonomy/Browser/wwwtax.cgi?id=176309) | 11.4% | 22.7% | 6.9% |
| [*Methanomassiliicoccaceae archaeon DOK*](https://www.ncbi.nlm.nih.gov/Taxonomy/Browser/wwwtax.cgi?id=1535962) | 28.6% | 6.8% | 0 |
| [*Methanobrevibacter woesei*](https://www.ncbi.nlm.nih.gov/Taxonomy/Browser/wwwtax.cgi?id=190976) | 37.1% | 43.2% | 86.2% |
| *Uncultured Archaeon* | 20% | 20.4% | 6.9% |
| *Methanogenic archaeon CH1270* | 2.8% | 0 | 0 |
| *Methanorbis furvi strain Ag1* | 0 | 6.8% | 0 |
